# Supplementary material for: Versatile CRISPR/Cas9 Systems for Genome Editing in Ustilago maydis
Source: J Fungi (Basel). 2021 Feb 18;7(2):149. doi: 10.3390/jof7020149 (PMC7922307; doi:10.3390/jof7020149)
Supplement: Supplementary file 1 [file jof-07-00149-s001.zip › Figure S2.pdf]

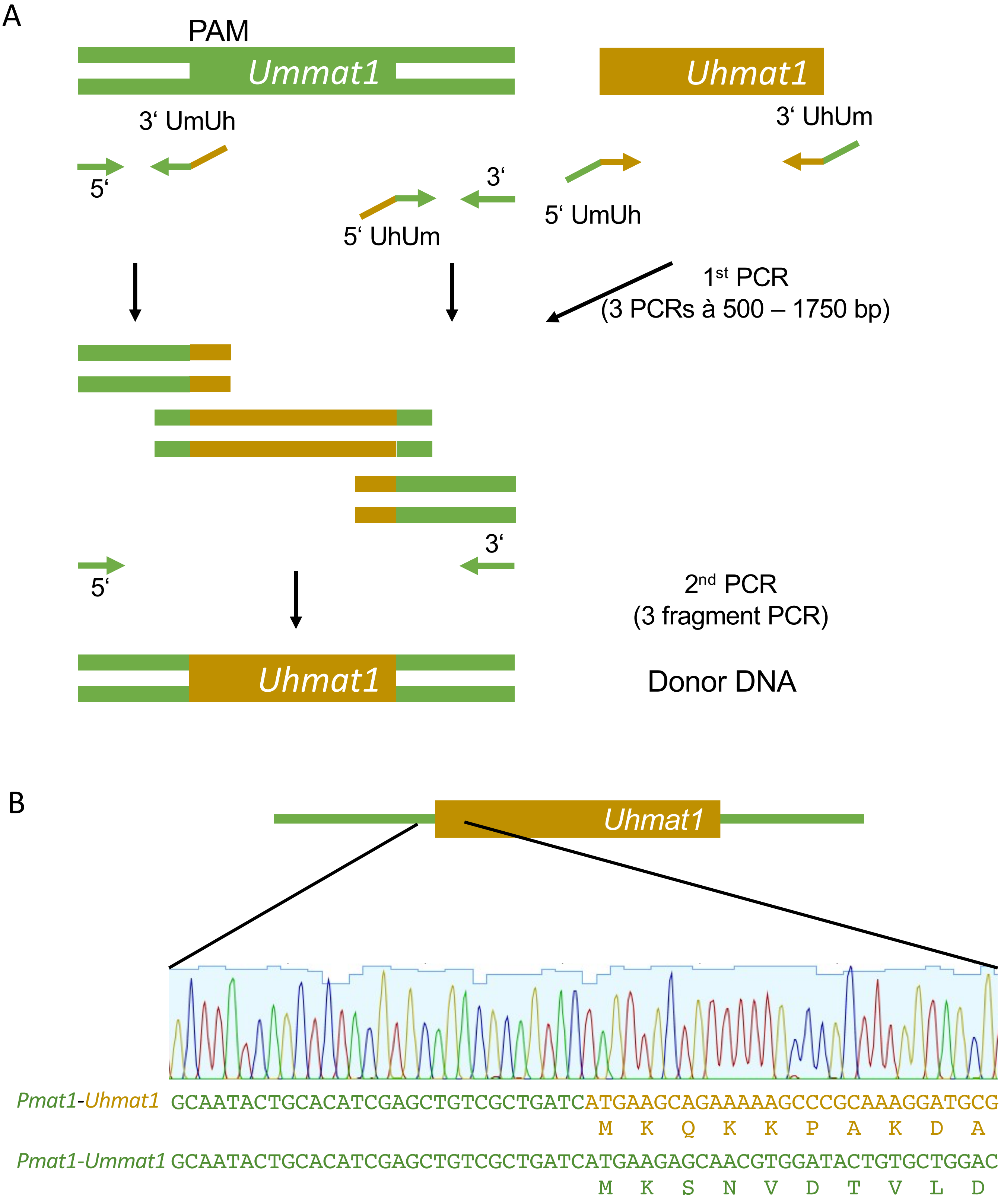
A
PAM
Ummat1
Uhmat1
3‘ UmUh
3‘ UhUm
5‘
3‘
5‘ UmUh
5‘ UhUm
1st PCR
(3 PCRs à 500 – 1750 bp)
3‘
5‘
2nd PCR
(3 fragment PCR)
Uhmat1
Donor DNA
B
Uhmat1
Pmat1-Uhmat1 GCAATACTGCACATCGAGCTGTCGCTGATCATGAAGCAGAAAAAGCCCGCAAAGGATGCG
M K Q K K P A K D A
Pmat1-Ummat1 GCAATACTGCACATCGAGCTGTCGCTGATCATGAAGAGCAACGTGGATACTGTGCTGGAC
M K S N V D T V L D
