# Supplementary material for: Versatile CRISPR/Cas9 Systems for Genome Editing in Ustilago maydis
Source: J Fungi (Basel). 2021 Feb 18;7(2):149. doi: 10.3390/jof7020149 (PMC7922307; doi:10.3390/jof7020149)
Supplement: Supplementary file 1 [file jof-07-00149-s001.zip › Wege et al_Table S1.docx]

**Supplementary Table S1**

| Strain | Reference | Plasmid transformed | Resistence | Progenitor strain |
| --- | --- | --- | --- | --- |
| *Ustilago maydis* MB215 | [60] |  |  |  |
| *Ustilago maydis* Bub8 | [59] |  |  |  |
| *Ustilago hordei* Uh4857-4 | [53] |  |  |  |
| Bub8 ∆*don3* |  | pSM2-Don3 + donor DNA |  | Bub8 |
| Bub8 *pho85as* |  | pSM2-Pho85 + donor DNA |  | Bub8 |
| MB215 ∆*rua1* | [44] |  | hyg | MB215 |
| MB215 ∆*rua1* ∆*mat1* | this study | pSM2-Mat1 | hyg | MB215 ∆*rua1* |
| MB215 ∆*rua1Uhmat1* | this study | pSM2-Mat1 + donor DNA | hyg | MB215 ∆*rua1* |
| MB215 *mCherry-fab4* | this study | pSM2-Fab4 + donor DNA |  | MB215 |
| MB215 *mCherry-fab4 Pmac1:GFP-mac1* | this study | pPmac1-GFP-Mac1 [40] | cbx | MB215 *mCherry-fab4* |
|  |  |  |  |  |

Strains used in this study. Note that it is important to loose the pCas9 plasmids by multiple rounds of growth on cbx-free plates after the identification of positive clones.
