# Supplementary material for: Versatile CRISPR/Cas9 Systems for Genome Editing in Ustilago maydis
Source: J Fungi (Basel). 2021 Feb 18;7(2):149. doi: 10.3390/jof7020149 (PMC7922307; doi:10.3390/jof7020149)
Supplement: Supplementary file 1 [file jof-07-00149-s001.zip › Wege et al_Table S3.docx]

**Supplementary Table S2**

| Plasmid | used for | cloning procedure |
| --- | --- | --- |
| pCas9_sgRNA_0 |  | [23] |
| pSM1 | CRISPR/Cas9 vector | see text below |
| pSM2 | CRISPR/Cas9 vector with Cas9*hf* | see text below |
| pSM2-Don3 | Deletion of *don3* | The annealed Primer pair MJ418/MJ419 was cloned in pSM2 digested with SnaBI and XbaI. |
| pSM2-Pho85 | Double strand break in the *pho85* gene | The annealed Primer pair Pho85_sgDNA fwd/ Pho85_sgDNA fwd was cloned in pSM2 digested with SnaBI and XbaI. |
| pSM2-Mat1 | Double strand break in the *mat1* gene | The annealed Primer pair MK883/MK884 was cloned in pSM2 digested with SnaBI and XbaI. |
| pSM2-Fab4 | Double strand break in the *fab4* gene | The annealed Primer pair ML314/M315 was cloned in pSM2 digested with SnaBI and XbaI. |
| pPmac1-GFP-Mac1 | Peroxisomal marker | [40] |
|  |  |  |
|  |  |  |

Plasmids used in this study.

**Construction of pSM1**

The complementary primers MJ388 and MJ389 were hybridized and used in a Gibson assembly reaction together with the linearized CRISPR/Cas9 plasmid pCas9_sgRNA_0 according to the protocol of [23].

**Construction of pSM2**

Site-directed mutagenesis to generate cas9hf in pSM2 was performed by Gibson assembly using four overlapping PCR products on *cas9* (MJ465 + MJ467; MJ466 + MJ469; MJ468 + MJ471; MJ470 + MJ471) together with pSM1 linearized with the restriction enzymes Bsu36I and FspAI.
