# Supplementary figures and images for: Versatile CRISPR/Cas9 Systems for Genome Editing in Ustilago maydis
Source: J Fungi (Basel). 2021 Feb 18;7(2):149. doi: 10.3390/jof7020149 (PMC7922307; doi:10.3390/jof7020149)

A

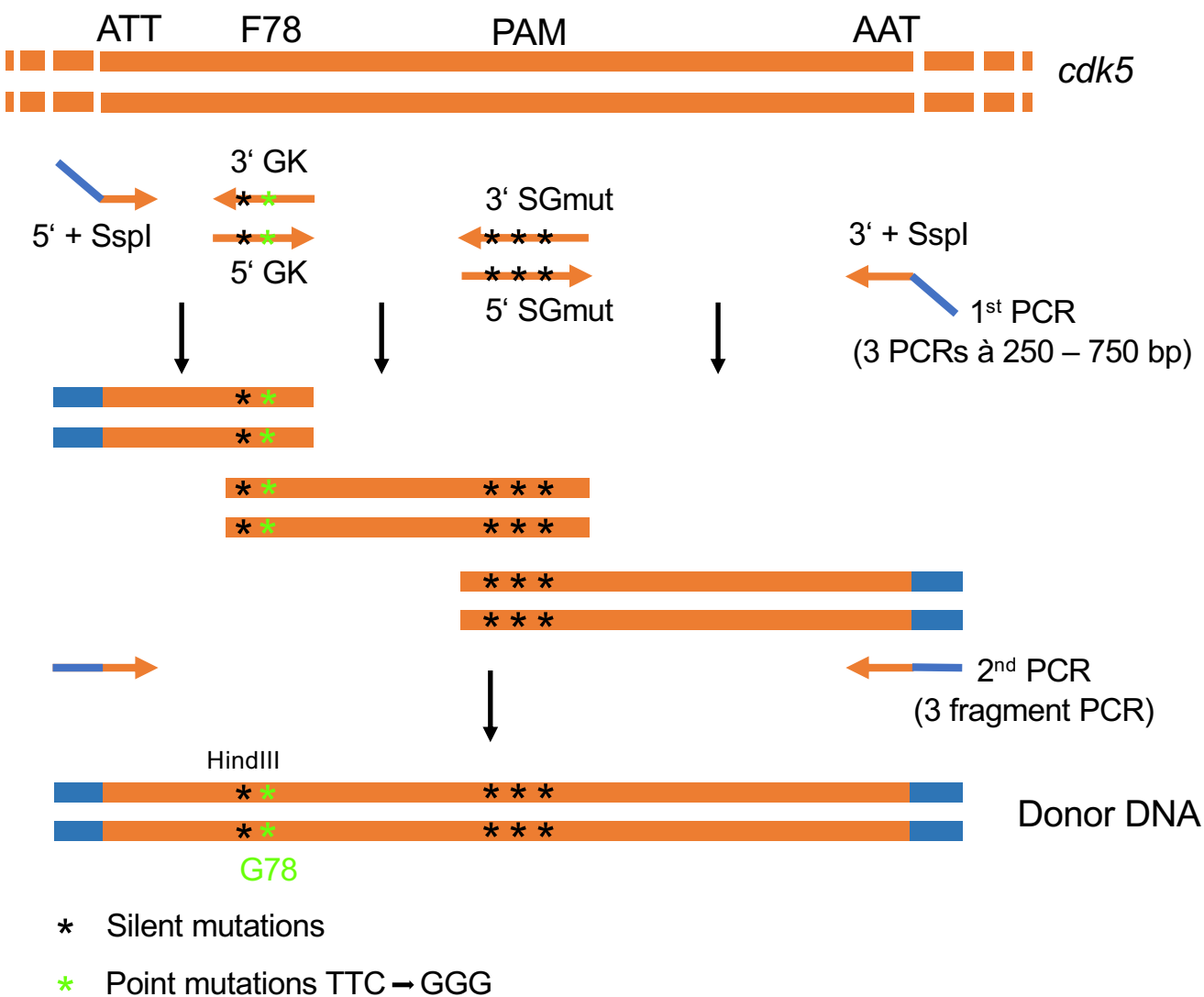

B

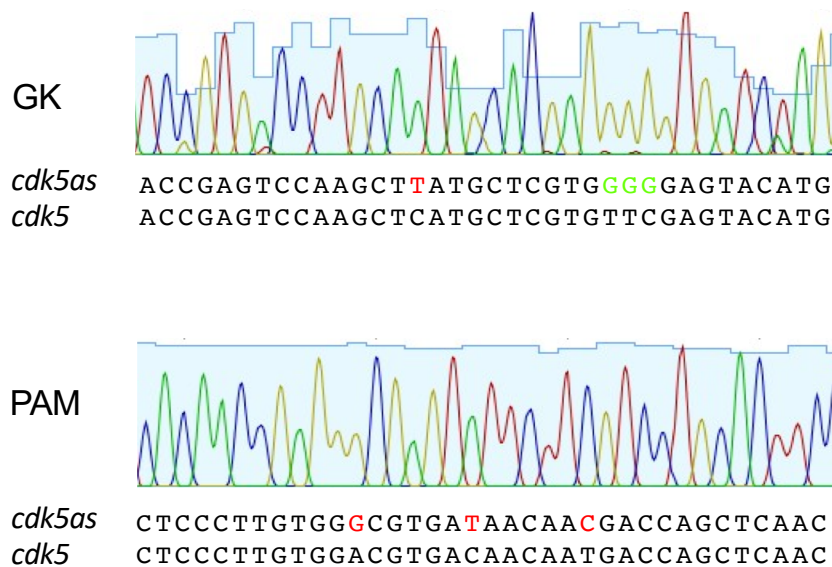

Supplement: Supplementary file 1 [file jof-07-00149-s001.zip › Figure S1.pdf]

A

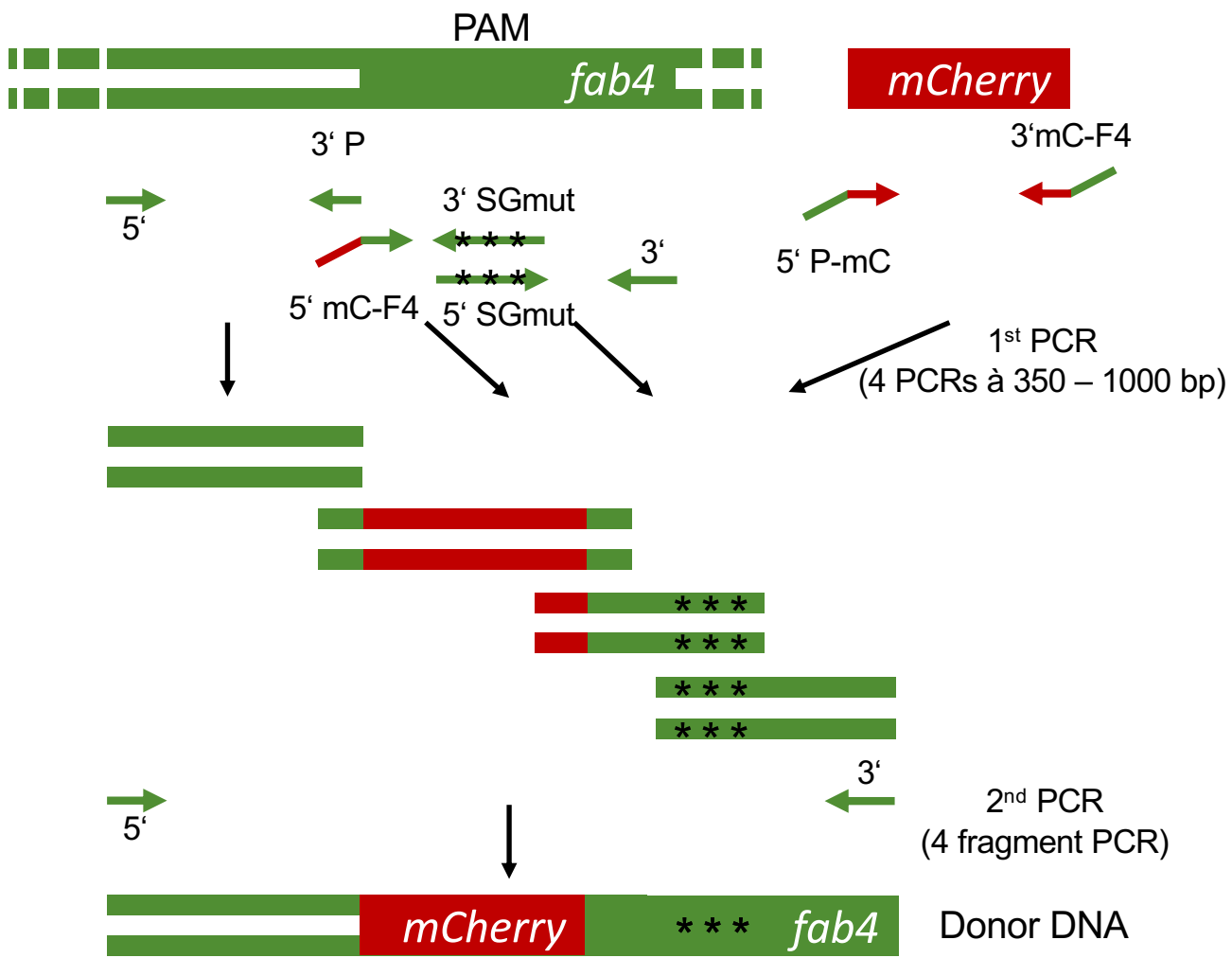

B

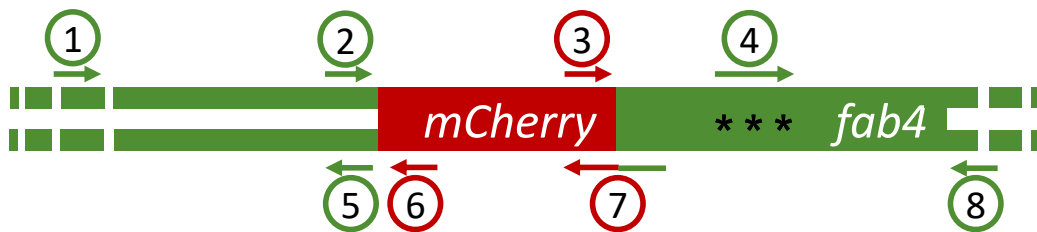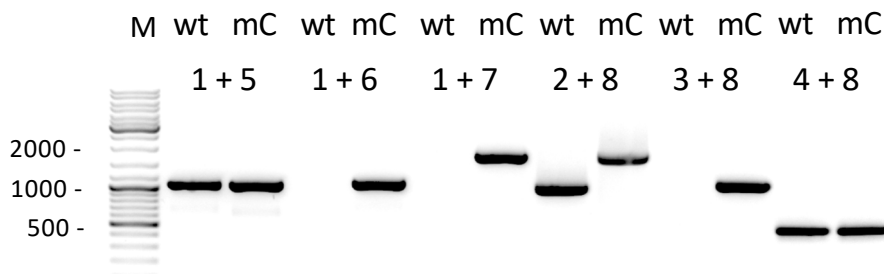

Supplement: Supplementary file 1 [file jof-07-00149-s001.zip › Figure S3.pdf]
